# Rheumatoid arthritis in Latin America: challenges and solutions to improve its diagnosis and treatment training for medical professionals

**DOI:** 10.1007/s10067-015-3033-8

**Published:** 2015-07-31

**Authors:** R. Munoz-Louis, J. Medrano-Sánchez, R. Montufar

**Affiliations:** Hospital Docente Padre Billini, Santo Domingo, Dominican Republic; Consultorio de Especialidades del Instituto Salvadoreño del Seguro Social, San Salvador, El Salvador

**Keywords:** Complete solutions, Difficulties in education, Medical professionals, Rheumatoid arthritis

## Abstract

Rheumatoid arthritis (RA) is a disease with multiple clinical manifestations and chronic complications that requires a multidisciplinary team to treat and monitor patients. This understanding between the different medical and health professionals is essential in obtaining patient well-being. This is the reason behind the assessment of the difficulties and limitations seen in Latin America in the field of rheumatology. The aim is to suggest possible mechanisms and solutions to strengthen the knowledge and understanding of the way the disease behaves and how it can be handled by doctors and medical professionals.

## Introduction

Rheumatoid arthritis (RA) is a chronic, disabling, painful disease. This means that very often patients are seen not only by a rheumatologist but also by other professionals, such as nurses, physiotherapists, social workers, psychologists and nutritionists. However, the potential collaboration of these different areas, which would enable the patients’ disease and quality of life to improve, is not as satisfactory as it should be. This is often due to the fact that these professionals are simply unaware of the possible benefits achieved through joint management of the problem.

Despite its high prevalence, there is a major lack of education, in both undergraduate and postgraduate training, about RA and how RA patients can be treated. This failing significantly damages the population suffering from this disease, as it forms a technical limit that makes early referral and suitable patient monitoring more difficult. In a multicentric study published by Burgos-Vargas et al., it was seen that the lack of GP training on musculoskeletal disorders is one of the obstacles in ensuring correct treatment of rheumatoid arthritis [1].

A full consultation provides the opportunity to handle RA patients using holistic care. The term is used meaning treatment that takes due consideration of the fact that a person is made up from a set of biophysical, social, cognitive and psychological factors that come together to form a whole that is greater than the sum of its parts, and which interact constantly. This means that when one part is affected, naturally, so are the others [2], and this is the reason why right from the start, and regularly, not only the disease but also other areas of importance to the patient also need to be assessed [3].

This document aims to describe the challenges faced by medical professionals in the field of rheumatology. It also seeks to propose strategies that can improve their training in handling rheumatoid arthritis patients.

### Challenges in the training of rheumatology medical professionals

We are seeing more and more convincing evidence of the success of multidisciplinary and interdisciplinary management of RA patients [4–7]. This means that with every day that passes, it becomes even more important to strengthen knowledge of RA, both amongst the multidisciplinary working teams and in undergraduate and postgraduate professional medical training programs.

Few studies have been published in Latin America to determine the real social, economic and psychological impact of the damages caused by RA. This can limit decision-making, as the effective impact on the direct and indirect costs of the disease, as well as the damage it causes, are underestimated. Data is available from countries like Colombia, where RA is the second greatest cause of loss of healthy years of life due to the disability in working age [8].

As part of the evaluation of the academic level of knowledge in undergraduate education, both in medicine and other degrees associated with the treatment of RA patients, the academic programs or syllabus of universities in the Dominican Republic and El Salvador were revised. It was found that in studying medicine, the number of course or credit units assigned to the rheumatology module was no more than 1 % of the total programme credits https://www.ues.edu.sv/descargas/catalogocompleto/Medicina.pdf.

In aiming to assess the level of knowledge of professionals monitoring and treating RA patients, a survey was carried out amongst the professionals of two Latin America countries (the Dominican Republic and El Salvador), evaluating 27 professionals working in both public and private practice (Table 1).

**Table 1:** Number of people interviewed and the average time spent in minutes by each of them to fill out the survey

Professions	No. of respondents per area	Average time spent on the survey (in minutes)	Average hours of RA class content in undergraduate studies	Knowledge of RA (good, acceptable, poor)	Classed by rheumatologist (yes/no)
Nurses	8	8	0–5	Acceptable	No
Psychologists	2	6	0–5	Acceptable	No
Social workers	4	10	0–5	Poor	No
Physiotherapists	8	7	0–5	Good	No
Health promoter	1	10	0–5	Poor	No
Nutritionist	4	6	0–5	Acceptable	No
Total	27	47			

We find the following: 100 % of respondents declared that they had received at least 5 h of class on RA during undergraduate training; none had received classes delivered by a rheumatologist. In an attempt to assess knowledge of the professionals about RA, they were asked to describe how they rated their knowledge of the disease (as good, acceptable or poor). It was found that 51.8 % (no., 14) reported it as acceptable, 29.6 % (no., 8) as good and just 18.5 % (no., 5) as poor.

No surveys were conducted amongst pharmacology graduates, as this profession, in our countries, has no direct relationship with patients; they are employed in administrative roles by the various hospitals.

To conclude, we should mention that few studies have been published in our countries to determine the real social, economic and psychological impact of the damages caused by RA. This can limit decision-making, as the effective impact on the direct and indirect costs of the disease, as well as the damage it causes, are underestimated. Data is available from countries like Colombia, where RA is the second greatest cause of loss of healthy years of life due to disability in working-aged people [8].

### Possible solutions in training medical professionals

The loss of the capacity to work caused by RA is extremely important as approximately half of cases begin during working age, and in the space of 10 years, between 26 and 60 % of patients find themselves forced to stop work [8, 9].

In accordance with that assessed in two Latin American countries, and extrapolating this data to cover other countries in the region, below we have chosen to deconstruct the role played by each profession in the treatment of RA patients. We will also make some suggestions for inclusion in the development of the undergraduate and postgraduate education programs of the different professions.

### Nurses

The role played by nurses in rheumatology is under development. In his work, Dr. De la Torre claimed that with the resurgence of the latest-generation drugs, like the biological agents, and the close monitoring they require, a debate had arisen on the role played by nurses in patient care [10]. Regarding a degree in nursing, and in accordance with the recommendations given in European guides on the handling of patients with chronic inflammatory arthritis [11], we believe that specialized courses or diplomas are necessary, teaching patient self-management strategies. Patient educational programs, knowledge of disease-modifying drugs and biological treatments and incident management by means of telephone consults should also be included and encouraged through the award of the title of “Specialist in joint inflammatory diseases”.

### Psychologists

We should note that the functional disability suffered by rheumatoid arthritis patients tends to isolate them from society. In turn, this forces them to endure various psychological and emotional processes such as negative feelings towards this disability, depression, which is an increased risk with respect to the general population, and stress, which depends on the time of disease evolution and may be present right from the start of the disease or appear after a relapse [12]. Depression is one of the most significant psychological consequences and frequently results in the use of psychotropic drugs [9]. In psychology, considering the fact that the survey reported “acceptable” knowledge about the disease, we would recommend that interdisciplinary events be organized. These should involve both rheumatologists and psychologists and discuss matters relating to psychological well-being, mood changes due to functional disability and absenteeism from work [13].

### Nutritionists

In assessing the nutritional area, we can see that the literature reports a great many uncontrolled studies, and some controlled studies, where an attempt has been made to evaluate the interaction of certain foods with RA activity. The prevalence of alterations in the nutritional state and the anthropometric parameters in RA patients has been described in various studies, in which a reduced quantity of lean body mass caused by the increased catabolism of structural proteins and consequent depletion of muscle, visceral and immune tissue are noted. These, in turn, end up causing “rheumatoid cachexia” [14, 15]. We suggest using nutritional evaluation as a key tool by which to classify patients according to malnutrition levels. We also recommend educational conversations or meetings organized for patients, social workers, health promoters and nurses in connection with the nutritional requirements of RA patients.

The general recommendations given by Pan-American League of Associations for Rheumatology (PANLAR) [16] suggest that planning meetings be held with universities offering academic programs for medical professionals on how to treat RA patients. The aim is to get rheumatologists involved in undergraduate training and thereby increase knowledge about the disease. Recommendations that could be introduced include increasing credit hours allocated to rheumatology, promoting scientific sessions, round tables and interdisciplinary symposiums.

In the same way, the creation of graduates in nursing, nutrition, psychology and physiotherapy can be stimulated, with a view to increasing knowledge and improving treatment of the RA patient.

We believe that regional awareness studies should be proposed through surveys and interviews conducted with medical professionals so that PANLAR can suggest possible solutions in medical professional training.

**Table:** 

Problems in training medical professionals	Suggested solutions in training medical professionals
Lack of nursing graduates specialized in the care of patients with inflammatory joint disease	Postgraduate nursing course specialized in inflammatory joint diseases
Undergraduate rheumatology course taught by a non-rheumatologist doctor	Include a rheumatology course taught by a rheumatologist in the undergraduate programme
Few undergraduate hours/classes	Increase the number of undergraduate hours/classes
Lack of epidemiological studies in Latin America and the impact of RA	Carry out studies on the impact of RA on employment, social and psychological aspects, in Latin America

## References

[1] Burgos-Vargas R, Catoggio LJ, Galarza-Maldonado C, Ostojich K, Cardiel MH (2013). Current therapies in rheumatoid arthritis: a Latin America perspective. Reumatol Clin.

[2] Erickson HL (2007). Philosophy and theory of holism. Nurs Clin North Am.

[3] de la Torre Aboki J (2011). Aportación de la consulta de enfermería en el manejo del paciente con artritis reumatoide. Rheumatol Clin.

[4] Marion CE, Balfe LM (2011). Potential advantages of interprofessional care in rheumatoid arthritis. J Manag Care Pharm.

[5] Illingworth P, Chelvanayagam S (2007) Benefits of interprofessional education in health care. Br J Nurs 16(2):121–124. doi:10.12968/bjon.2007.16.2.22773 10.12968/bjon.2007.16.2.22773 17353824

[6] Vliet Vlieland TP (2004). Multidisciplinary team care and outcomes in rheumatoid arthritis. Curr Opin Rheumatol.

[7] Vliet Vlieland TP, Hazes JM (1997). Efficacy of multidisciplinary team care programs in rheumatoid arthritis. Semin Arthritis Rheum.

[8] de Salud M (1998). Dirección de Estudios Económicos e Inversión Publica de Minsalud.

[9] García-Vadillo JA, Castañeda S, Carrasco AL, Jimeno A (2001). Costes económicos de la artritis reumatoide de corta evolución. Rev Esp Reumatol.

[10] DE LA TORRE J, HILL J (2008). Desarrollo y momento actual de la Enfermería reumatológica.

[11] Van Eijk-Hustings Y, Van Tubergen A, Boströn C (2012). EULAR recommendations for the role of the nurse in the management of chronic inflammatory arthritis. Ann Rheum Dis.

[12] Quiceno JM, Vinaccia S (2011). Rheumatoid arthritis: psychobiological considerations. Divers Perspect Psicol.

[13] Keefe FJ, Smith SJ, Buffington AL (2002). Recent advances and future directions in the biopsychosocial assessment and treatment of arthritis. J Consult Clin Psychol.

[14] Walsmith J, Roubenoff R (2002). Cachexia in rheumatoid arthritis. Int J Cardiol.

[15] Gómez-Vaquero C, Nolla J, Fiter J, Ramon JM, Concustell R, Valverde J (2001). Nutritional status in patients with rheumatoid arthritis. Joint Bone Spine.

[16] Massardo L, Suarez-Almazor ME, Mario H, Massardo L, Suarez-Almazor ME, Cardiel MH (2009). Management of patients with rheumatoid arthritis in Latin America: a consensus position paper from Pan-American League of Associations of Rheumatology and Grupo Latino Americano De Estudio de Artritis Reumatoide. J Clin Rheumatology.

